# Movement Velocity as A Measure of Exercise Intensity in Persons with Multiple Sclerosis: A Validity Study

**DOI:** 10.3390/jcm9082458

**Published:** 2020-07-31

**Authors:** Luis Andreu-Caravaca, Domingo Jesús Ramos-Campo, Oriol Abellán-Aynés, Jacobo Ángel Rubio-Arias

**Affiliations:** 1 Faculty of Sports, UCAM, Catholic University San Antonio, 30107 Murcia, Spain; landreu@ucam.edu (L.A.-C.); djramos@ucam.edu (D.J.R.-C.); oabellan@ucam.edu (O.A.-A.); 2 International Chair of Sport Medicine, UCAM, Catholic University San Antonio, 30107 Murcia, Spain; 3 LFE Research Group, Department of Health and Human Performance, Faculty of 15 Physical Activity and Sport Science-INEF, Universidad Politécnica de Madrid, 28040 16 Madrid, Spain

**Keywords:** bench press, leg press, 1RM prediction, velocity-based training, neurological disorders

## Abstract

Objectives: This study aims to analyse the validity (agreement between two methods) of the movement propulsive velocity (MPV) as an indicator of relative load in leg press (LP) and bench press (BP) exercises in persons with multiple sclerosis (MS). Methods: 18 persons with MS (sex = 55% male; age (mean ± SD) = 44.88 ± 10.62 years; body mass = 67.19 ± 10.63 kg; height = 1.66 ± 0.07 m; Expanded Disability Status Scale (EDSS) = 3.12 ± 1.73) performed an incremental loading test in BP and LP exercises in two separate sessions. Individual determination of the one-repetition maximum (1RM) and full load-velocity profile were obtained for each participant. Results: a significant linear relationship was observed between the %1RM load and the MPV in LP (%1RM = −133.58 × MPV + 117.44; r^2^ = 0.84; standard error of the estimate (SEE) = 9.38%1RM) and BP (%1RM = −95.66 × MPV + 115.26; r^2^ = 0.86; SEE = 9.82%1RM). In addition, no significant differences were found between the %1RM achieved directly and the %1RM obtained by the equation calculated from the linear regression (LP, *p* = 0.996; BP, *p* = 0.749). Conclusions: these results indicate that movement velocity can estimate the relative load in bench press and leg press exercises in persons MS.

## 1. Introduction

Persons who have multiple sclerosis (MS), an autoimmune disorder that leads to myelin and axon loss, present a variety of symptoms, such as spastic paresis [1], impaired balance [2], and ventilatory muscle weakness [3]. Additionally, persons with MS have reduced muscle strength, which can be due to lower neuromuscular activation [4] or lower physical activity and (or) sedentarism [5]. However, these consequences mentioned above have been shown to improve with the practice of individualised physical activity and exercise [6].

Resistance training in MS has shown not only improvements in muscle strength or functional capacity [7], but also reduced fatigue, better mood, and quality of life [8]. In addition, these increases in lower extremity muscle strength have been associated with increased quality of life in people with MS [8]. Thus, the optimisation of strength training programs is one to achieve improved strength and power. In this way, manipulation of strength training variables, in particular, the intensity of training loads within a periodical program is a key factor in maximising strength training gains [9,10]. In this respect, control of resistance training adaptations to individualise the training loads usually uses the one-repetition maximum test (1RM) [11] since 1RM is the main variable used to control resistance training loads [12]. The problem with the direct calculation of 1RM is related to the time and energy-consumption required for the assessment [13]; along with increments of fatigue related to the lack of muscle strength and power in MS patients [14].

Additionally, MS patients’ conditions may vary daily because of environmental effects, such as temperature [15,16]; this fact indicates that training intensities must be assessed frequently using methods that do not cause high muscle fatigue or high energy requirements. Thus, methods other than 1RM should be performed to control training loads in different sessions. A direct assessment of 1RM has some adverse effects, including fatigue, mainly due to the high number of series to be carried out. This occurs with healthy people, so logically, in persons with MS and where fatigue is a symptom, direct assessment is not recommended [17]. In addition, performing a direct assessment of 1RM increases the injury risk and stress on the muscles and joints, mainly in untrained people [18] and clinical populations [19]. Moreover, resistance training causes gains of strength in MS patients [7]; hence, 1RM should be assessed regularly to adapt the training loads to the strength improvements.

Mean propulsive velocity (MPV) has been used to estimate the 1RM due to its high linear correlation with the 1RM in different exercises, e.g., bench press [20] or leg press [21] in trained individuals and athletes. These linear relationships have been shown in older women for both bench and leg press exercises [22]. This assessment method for 1RM might be very interesting and recommendable in individuals suffering from MS to indirectly calculate the 1RM for the control of the training load. As MS patients have lower muscle strength and power compared to a healthy population [14], different MPV values may appear in lower external loads, and these equations could be inaccurate for MS patients. However, no previous research has evaluated 1RM in MS patients. Consequently, the main aim of this project was to determine the direct relationship between MPV and the relative 1RM load (%1RM) in two different multi-joint exercises, one of the upper-body (bench press) and one of the lower-body (leg press) in MS patients. Consequently, we hypothesized that mean propulsive velocity would be a valid and reliable method for estimating the maximum strength (1RM) in bench press and leg press in persons with MS.

## 2. Methods

### 2.1. Participants

A total of 18 persons with MS were recruited from the local MS association (Table 1). All participants were previously diagnosed with MS by a board-certified neurologist according to the McDonald criteria [23]. Inclusion criteria were: (a) diagnosed with relapsing-remitting MS, (b) had a mild or moderate disability with clinical mild spastic-ataxic gait disorders, (c) was in the stable phase of the disease, and (d) had previous experience with the bench press and leg press exercises. The exclusion criteria were: (a) EDSS >6, (b) relapse within the preceding six months and (c) musculoskeletal injuries or physical limitation that could affect tested performance, and (d) corticoid treatments 28 days before the study start. Two participants did not perform the bench press and the leg press testing session, respectively. Therefore, the load-velocity relationship was determined for 17 persons with MS in each exercise. The present study was approved by the Catholic University of Murcia’s Science Ethics Committee (CE071923) and was performed under the Declaration of Helsinki. All participants provided written and signed informed consent before starting the study.

### 2.2. Study Design

All training and testing sessions were completed in the UCAM (Universidad Católica San Antonio de Murcia) Sports Center (Murcia, Spain). A cross-sectional study was developed to analyse whether movement velocity could be a valid and reliable method for estimating the percentage of 1RM (relative load) during the leg press and bench press exercises in persons with MS. The chosen exercises were the bench press and leg press as two of the most important exercises for improving both upper and lower body strength [7,24]. Participants had three familiarisation sessions with leg press and bench press exercises to perform the exercises with an appropriate technique. Participants returned one week later for performing the leg or bench press testing session, and 72 h later, participants performed the other exercise in a randomised order. Sessions were completed at the same time of the day for each participant and under the same environmental conditions. The bench press and leg press exercises were completed in different sessions in a randomised order. The load-velocity relationship was determined in the two testing sessions using a standard incremental loading test that has been described in other studies [21,25]. All sessions were supervised by the same experienced researcher, who ensured that all participants exerted maximum effort.

### 2.3. Leg Press Testing Procedure

Initially, a standardised warm-up was performed that included five minutes on a cycloergometer, followed by dynamic stretching of the lower-body and one set of 10 repetitions against 10 kg during the leg press exercise. The participants started with an external load of 30 kg that was increased until they reached the maximum load that they were able to lift in 1RM. The load increases depended on the mean propulsive velocity (MPV) achieved during the set, following the recommendations of previous studies [22]: MPV > 0.80 m × s^−1^ determined load increase of 20 kg; 0.80 m × s^−1^ ≥ MPV ≥ 0.30 m × s^−1^ determined load increase of 10 kg; and MPV < 0.30 m × s^−1^ determined load increase of 5 kg. Participants performed three repetitions with light loads, two repetitions with medium loads, and one repetition with heavy loads. Light loads were considered (MPV > 1.00 m×s^−1^), medium loads (1.00 m × s^−1^ ≥ MPV ≥ 0.45 m × s^−1^) and heavy loads (MPV < 0.45 m × s^−1^). Participants performed three repetitions with light loads, two repetitions with medium loads, and one repetition with heavy loads. Five minutes were given to rest between sets. The highest MPV of each load was analysed. To perform a correct measurement of the MPV, the eccentric phase of the movement lasted 2 s and was controlled by a metronome, until the participant reached 90° of knee flexion, position in which a stop was placed so that knee flexion of more than 90° would not occur. In this position, a concentric phase was performed as fast as possible. Participants performed an average of 9.23 ± 4.54 sets. A horizontal leg press machine (Technogym, Cesena, Italy) was used for the study. To measure the MPV of the movement, a linear position transducer (Chronojump, Barcelona, Spain) was used. The device was fixed to the machine platform.

### 2.4. Bench Press Testing Procedure

Initially, a standardised warm-up was performed with five minutes on an arm ergometer, followed by dynamic stretching of the upper-body and one set of 10 repetitions against 5 kg (mass of the unloaded barbell) during the bench press exercise. The participants started without an external load (only with the barbell) and the external load was added until they reached the maximum load that they were able to lift in 1RM. The load increases depended on the MPV achieved during the set: MPV > 0.80 m × s^−1^ determined load increase of 5 kg; 0.80 m × s^−1^ ≥ MPV ≥ 0.30 m × s^−1^ determined load increase of 2.5 kg; and MPV < 0.30 m × s^−1^ determined load increase of 1 kg. Participants performed three repetitions with light loads, two repetitions with medium loads, and one repetition with heavy loads. Light loads were considered (MPV > 1.00 m × s^−1^), medium loads (1.00 m × s^−1^ ≥ MPV ≥ 0.45 m×s^−1^) and heavy loads (MPV < 0.45 m × s^−1^). Five minutes were given to rest between sets. The highest MPV of each load was analysed. To make a correct measurement of the MPV, the eccentric phase of the movement was controlled until the barbell was in contact with the chest. In this position, a concentric phase was performed as fast as possible until their elbows reached full extension. Participants performed an average of 10.21 ± 4.56 sets. A Smith machine (Technogym, Cesena, Italy) was used for this exercise. To measure the MPV of the movement, a linear position transducer (Chronojump, Barcelona, Spain) was used. The device was fixed to the left side of the barbell.

### 2.5. Statistical Analyses

The statistical analyses were conducted using Jamovi. Available online: jamovi.org (accessed on 15 March 2020) (Jamovi Project 2018, version 0.9.1.7) and statistical package SPSS (version 22.0: SPSS, Inc., Chicago, IL, USA) for Windows. Before the data analysis, the Kolmogorov-Smirnov test was used to determine the normal distribution of the variables. The relationship between relative load (%1RM) and MPV were determined by linear regression models (first-order polynomials). The relationships were initially assessed by Pearson’s coefficient correlation (*r*). The magnitude of the correlations was assessed according to Hopkins et al. [26]. The goodness of fit was tested by the Pearson multivariate determination coefficient (r^2^), F-statistic, and error of estimation (SEE) [27]. Subsequently, the MPV was calculated using the obtained equations for each participant and the individual results were shown for each of the loads relative to the 1RM. In addition, to analyse the level of agreement (reliability) between the direct %1RM and the %1RM obtained from the linear regression equation, the intraclass correlation coefficient (ICC; absolute agreement, two way random) was used. Threshold values for ICC reliability were <0.5 (poor), between 0.5 and 0.75 (moderate), between 0.75 and 0.9 (good), and ≥0.9 (excellent) [28]. Bland-Altman pairwise comparisons were used to evaluate whether there was an agreement or bias between the direct %1RM and the %1RM obtained from the linear regression equation [29]. Differences between methods (1-RM vs. 1-RM equation) were also tested with a paired t-test. Statistical significance was accepted at an alpha level of ≤0.05.

## 3. Results

**Figure 1:**
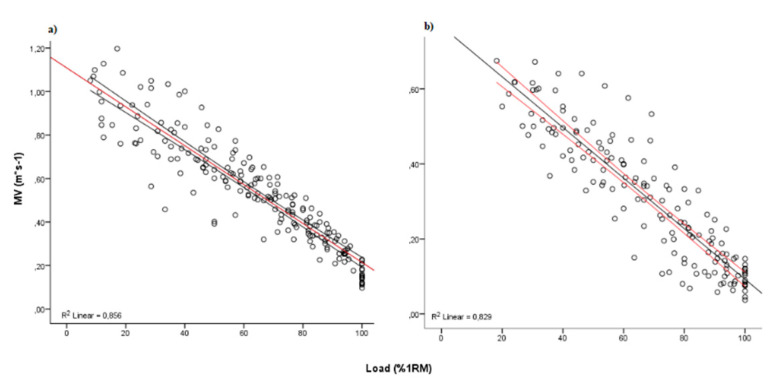
Relationship between training load (%1RM) and mean propulsive velocity (MPV) obtained during (**a**) bench press and (**b**) leg press.MV: Mean Velocity, R^2^: Pearson’s multivariate coefficient of determination.

A significant linear relationship was observed between the %1RM load and the MPV in both exercises (leg press, *n* = 156, *r* = −0.911, *p* < 0.001; bench press, *n* = 195, *r* = −0.925, *p* < 0.001). Additionally, a statistically significant linear regression model was observed (leg, *F* = 746.7, *p* < 0.0001; bench press, *F* = 1145.4, *p* < 0.0001). The linear regression model showed the following regression equations stimate the %1RM from MPV in people with MS (Figure 1):

Leg press %1RM = −133.58 × MPV + 117.44 (*r*^2^ = 0.84, SEE = 9.38%1RM)

Bench press %1RM = −95.66 ×MPV + 115.26 (*r*^2^ = 0.86, SEE = 9.82%1RM)

The values of the MPV at each 10% load increment in the leg press and bench press are shown in Table 2.

There were no significant differences between the %1RM obtained directly and the %1RM obtained by the equation obtained from the linear regression (Table 3).

**Figure 2:**
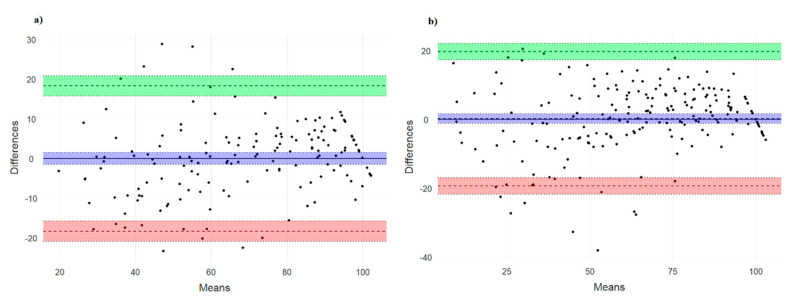
Altman plots representing the Bland central line and 95% limits of agreement between two methods. (**a**) leg press; (**b**) bench press.

The Bland-Altman graphs showed agreement between the two compared methods (Figure 2 and Table 4).

In addition, the mean difference between the two methods was not statistically different from 0 (leg press, t = −0.005; df = 153, *p* = 0.996; bench press, t = 0.320, *p* = 0.749). Moreover, Intra-class correlations showed an excellent mean absolute agreement (leg press, ICC (intraclass correlation coefficient) = 0.915, CI = 0.886–0.938; bench press, ICC = 0.919, CI = 0.894–0.938).

## 4. Discussion

This study aimed to determine the direct relationship between MPV and the relative 1RM load in upper and lower-body resistance exercises in MS patients. Previous research has shown that MPV is a valid and reliable method to accurately estimate %1RM without the need to directly assess the 1RM [20,21], avoiding the problems of direct measurement. However, these conclusions have been demonstrated in young and healthy individuals [30,31] or elder women [22], but not in populations suffering from a neurological disease.

The main finding of this project was that there is indeed a linear relationship between the external load in resistance exercise, confirming the study hypothesis. Furthermore, the MPV in both the upper and lower body, and the results calculated from the equation, are very close to a direct method of 1RM assessment that is considered to be valid and reliable. Interestingly, our results indicate that the MPV results associated with each %1RM are slower than those reported previously for young individuals, with the greatest difference in the lower limb exercise (leg press).

Regarding the precision of the load-velocity relationship, previous studies in young individuals reported a strong association in the bench and leg press (*r*^2^ ≥ 0.94) [20,21,25,31]. However, the relationship reported in our study was lower (*r*^2^ = 0.84 and SEE = 9.4% 1RM in leg press; *r*^2^ = 0.86 and SEE = 9.8% 1RM in the bench press) than previous studies with young individuals. Marcos-Pardo et al. [22] found a weaker association of load-velocity in the bench press (*r*^2^ = 0.83) and leg press (*r*^2^ = 0.91) and a lower MPV associated with a given %1RM in older women in comparison with young individuals. This fact could be related to the impairment in power production in ageing individuals [32]. Therefore, the relationship observed in this study is similar to that described by Marcos-Pardo et al. [22], indicating that movement velocity provides useful information for monitoring and designing resistance training programs for MS patients. However, patients, coaches, and physicians should be aware that the MPV results associated with each %1RM reported previously in older adults or young participants cannot be extrapolated to persons with MS. Thus, the estimation of %1RM from MPV in MS patients suggests that individualised training using the load-velocity relationship is needed.

Most training programs in MS populations involve lower limbs [6]. This fact can be explained in part by the proximity of lower limb strength and gait and quality of life in people with MS [33,34], as well as by the greater weakness of persons with MS in the lower limbs compared to the upper limbs [14]. For this reason, the leg press was one of the exercises included in the present study. Previous studies that analyzed the lower limb exercise found a strong load-velocity association in athletes [21] (*r*^2^ = 0.96) and older women [22] (*r*^2^ = 0.91). In this sense, the relationship reported here was weaker (*r*^2^ = 0.84; SEE = 9.4%) than the previous results.

On the other hand, the bench press is a common exercise in resistance training routines for healthy people [35] and persons with neurological disorders [36]. Therefore, the analysis of the load-velocity for control and monitor the intensity of this exercise shows a strong relationship between %1RM and movement velocity (*r*^2^ = 0.94–0.97) in young individuals [20,25,30,37]. The strength of the load-velocity relationship reported here was lower (*r*^2^ = 0.86) than the results in healthy people obtained previously. Nevertheless, our results are in agreement with a recent study with older women (*r*^2^ = 0.83) [22]. One possible factor that might affect the accuracy of the load-velocity relationship is the number of familiarisation sessions carried out before the training program [38]. Hence, it is recommended to include some familiarisation sessions to obtain an accurate association, especially in patients with low previous experience in this type of exercise.

Moreover, MPV results with each %1RM were slower than those reported previously for young individuals [21] and closer to those for older people [22]. Interestingly, these differences between relative load and velocity were higher (slower velocities) in leg press exercise. In this way, one possible factor that can modify the MPV associated with each relative load (%1RM) is the symptoms of the MS, because MS patients have a diminished ability to perform both static and dynamic muscle contractions [39]. Due to the incidence of the disease, the lower limbs are weaker in the ability to develop strength compared to the upper limbs [40]. The mechanism underlying the identified deficit has both neural and muscular components, as well as a loss of muscle mass [39], differences in the distribution of muscle fiber compared to healthy control [41] or reduced capacity to activate motor units, which mainly appears in lower-limbs muscles [42]. In addition, the ability to produce force in short periods is reduced mainly due to the impairment of neural origin [43]. Therefore, the lower velocities against each relative load observed in our study for MS patients could be explained by the effects of MS suggesting that this disease produces a higher impairment in power production and maximal strength affecting the load-velocity relationship.

## 5. Limitations and Strengths of the Study

We acknowledge some limitations which should be considered for data interpretation. The main limitation was the small number of persons with MS, which took part in the research. Another limitation is that the standard error of the estimate is greater than ideal in both exercises. Furthermore, another limitation was that the study included both sexes (men and women), while previous research suggested that sex could modify the precision of the % 1RM estimate [37]. Another limitation is that this study only included persons with RRMS and EDSS <6, so the results can only be generalized for this specific subgroup only. Furthermore, persons with MS obtained lower velocities in lower limb exercise (leg press) for each relative load (%1RM) than described previously for young individuals. Finally, practical recommendations should be restricted to MS patients with EDSS <6. However, more information about the response to training and the monitoring of resistance training exercise is required for MS patients.

## 6. Conclusions

MPV can be used to prescribe and control the load (%1RM) during resistance training sessions in persons with MS and a disability status <6. Therefore, the MPV is a reliable and valid method. These findings may help coaches and researchers who want to monitor the training load in persons with multiple sclerosis. Therefore, the main strength of the present study is the novelty of the study and the practical application of the results to the real clinical field. Physicians and coaches should acknowledge a very close relationship of movement velocity and %1RM that can be used as a training control in MS patients, avoiding the problems associated with direct assessment of 1RM (e.g., fatigue and possible symptoms exacerbation and time).

## Figures and Tables

**Table 1:** Participant characteristics.

Characteristics	Mean ± SD (*n* = 18)
Age (Years)	44.88 ± 10.62
Sex (Men:Women)	10:9
EDSS	3.12 ± 1.73
Weight (kg)	67.19 ± 10.63
Height (m)	1.66 ± 0.07
BMI (kg·m^−2^)	23.28 ± 5.79

SD: Standard Deviation, BMI: Body Mass Index, EDSS: Expanded Disability Status Scale.

**Table 2:** Predicted mean propulsive velocity (m·s^−1^) for the leg press and bench press at each intensity (%1RM).

	Bench Press	Leg Press
%1RM	MPV ± SD	MPV ± SD
20	0.90 ± 0.16	0.68 ± 0.10
30	0.82 ± 0.15	0.62 ± 0.09
40	0.73 ± 0.13	0.55 ± 0.08
50	0.64 ± 0.11	0.48 ± 0.07
60	0.55 ± 0.09	0.42 ± 0.06
70	0.46 ± 0.08	0.35 ± 0.05
80	0.37 ± 0.06	0.29 ± 0.05
90	0.29 ± 0.05	0.22 ± 0.04
100	0.20 ± 0.04	0.15 ± 0.04

MPV: Mean Propulsive Velocity, SD: Standard Deviation, 1RM: One-Repetition Maximum.

**Table 3:** T-test of paired samples for the difference between methods.

										95% CI		
	Mean %1RM	SD %1RM	Mean Equation %1RM	SD Equation %1RM	Statistic	df	*p*	Mean Difference	SE Difference	Lower	Upper	Cohen’s d
Leg Press	68.1	23.61	68.1	21.7	0.00522	153	0.996	0.004	0.754	−1.485	1.493	0.000
Bench Press	63.7	25.8	63.5	23.7	0.32	194	0.749	0.229	0.714	−1.179	1.636	0.023

CI: Confidence Interval, df: degrees of freedom, SD: Standard Deviation, SE: Standard Error, 1RM: One-Repetition Maximum.

**Table 4:** Bland-Altman differences between methods.

	Leg Press (*n* = 156)			Bench Press (*n* = 195)		
		95% CI			95% CI	
	Estimate	Lower	Upper	Estimate	Lower	Upper
Bias	−0.004	−1.493	1.485	0.229	−1.179	1.636
Lower limit of agreement	−18.335	−20.885	−15.785	−19.305	−21.715	−16.895
Upper limit of agreement	18.327	15.777	20.877	19.762	17.353	22.172

CI: Confidence Interval.
